# TBpore cluster: A novel phylogenetic pipeline for tuberculosis transmission studies using nanopore next-generation sequencing data

**DOI:** 10.1371/journal.pone.0325914

**Published:** 2025-06-16

**Authors:** Sophie Gagnon, Emmanuelle Ametepe, Floriane Point, William Cloutier Charette, Arpita Chakravarti, Paul Rivest, Pierre-Marie Akochy, Hafid Soualhine, Zamin Iqbal, Michael B. Hall, Simon Grandjean Lapierre

**Affiliations:** 1 Immunopathology Axis, Centre de Recherche du Centre Hospitalier de l’Université de Montréal, Montréal, Québec, Canada; 2 Department of Microbiology, Infectious Diseases and Immunology, Université de Montréal, Montréal, Québec, Canada; 3 École de Santé Publique de l’Université de Montréal, Université de Montréal, Montréal, Québec, Canada; 4 Direction de Santé Publique, Centre Intégré Universitaire de Soins et de Services Sociaux du Centre-Sud-de-l’Île-de-Montréal, Montréal, Québec, Canada; 5 Laboratoire de Santé Publique du Québec, Montréal, Québec, Canada; 6 National Microbiology Laboratory, Winnipeg, Canada; 7 European Molecular Biology Laboratory, European Bioinformatics Institute (EMBL-EBI), Hinxton, Cambridgeshire, United Kingdom; Khoo Teck Puat Hospital, SINGAPORE

## Abstract

**Background:**

Molecular typing of *Mycobacterium tuberculosis* complex isolates enhances understanding of tuberculosis (TB) transmission dynamics, supporting public health efforts in outbreak investigations. This study aims to validate TBpore, a novel bioinformatic pipeline for clustering TB transmission isolates using Oxford Nanopore Technology (ONT) data and comparing it against conventional Mycobacterial Interspersed Repetitive-Unit Variable Number (MIRU-VNTR) typing and Illumina sequencing.

**Methodology/Principal findings:**

This retrospective case-control study included 58 clinical isolates from two TB outbreaks in Canada, previously characterized by public health investigations and MIRU-VNTR typing. DNA extraction and sequencing were performed on both Illumina and ONT platforms. Illumina data were processed using Clockwork and psdm, while Nanopore data were analyzed with TBpore. SNP distances were used to compare clustering results across methods, with clusters defined by SNP distance thresholds of ≤5 and ≤12. Both sequencing methods showed a high degree of concordance in clustering results. All isolates from the *M. africanum* outbreak clustered within the defined SNP thresholds, consistent with MIRU-VNTR and epidemiological data. In the *M. tuberculosis* outbreak, 20 out of 21 isolates clustered similarly across methods, with one exception. Within outbreak pairwise SNP distances were lower with Nanopore.

**Conclusion/Significance:**

ONT sequencing and the TBpore pipeline offer an accurate alternative to Illumina technology for TB molecular epidemiology. This study suggests potential increased clustering sensitivity with Nanopore technology, warranting further validation on larger datasets with robust epidemiological metadata.

## Introduction

*Mycobacterium tuberculosis* complex molecular typing can improve our understanding of disease transmission dynamics and hence support public health systems in outbreak investigations and contact tracing [1]. *Mycobacterium tuberculosis* (TB) whole genome sequencing (WGS) is progressively replacing previous PCR-based molecular typing methods including MIRU-VNTR due to its increased resolution [2]. WGS is hence already implemented in a systematic and prospective approach in several public health laboratories (e.g., England, Netherlands, New York State) [3,4].

Oxford Nanopore Technology (ONT) next-generation sequencing (NGS) platforms, including the portable MinION sequencer, are increasingly used in TB diagnostics. *Hall et al.* recently compared ONT-based TB sequencing to the benchmark reference Illumina technology for its ability to predict drug resistance and cluster genomically related isolates [5]. This sequencing platform head-to-head comparative analysis did not include clinical isolates for which person-to-person transmission or an epidemiological outbreak context had been confirmed by conventional clinical and epidemiology investigations.

In this study, we report on the clinical validation of TBpore, a new publicly available bioinformatic analysis pipeline enabling molecular clustering of putative transmission isolates. TBpore is designed as a companion tool to the ONT compatible genotypic drug susceptibility testing pipeline Mykrobe enabling simulataneous drug resistance and clustering testing [6]. This is a retrospective case-control molecular epidemiology study to measure the clustering ability and resolution of TBpore compared to conventional MIRU-VNTR and established Illumina NGS-based molecular typing methods. This study includes isolates from domestic TB outbreaks in Canada, which were previously well characterized by routine clinical and public health investigations [7].

## Results

In the *M. africanum* outbreak, both WGS technologies and pipelines identified 4 isolates as lineage 5 (L5) and 12 isolates as lineage 6 (L6) using SNP-typing. Four L6 isolates had identical MIRU-type (2 2 4 4 2 2 2 4 3 5 2 2 3 5 4 5 6 2 4 4 3 3 2 4) and among those, respectively, three (MB066247, MB077499, MB086368) and one (MB085690) had a strong and moderate probability of being epidemiologically related according to field investigations [7]. In the *M. tuberculosis* lineage 4 (L4) outbreak, 21 isolates shared a MIRU-type (2 2 4 3 2 5 1 4 3 3 2 5 2 3 4 5 3 4 4 2 3 4 7 3). All patients were from the same Montreal area and shared one or more risk factors, including alcohol and/or drug use, supervised drug injection sites visits, homelessness or prostitution. In this putative outbreak context, it was not possible to establish direct nominal epidemiological links between all patients.

**Fig 1:**
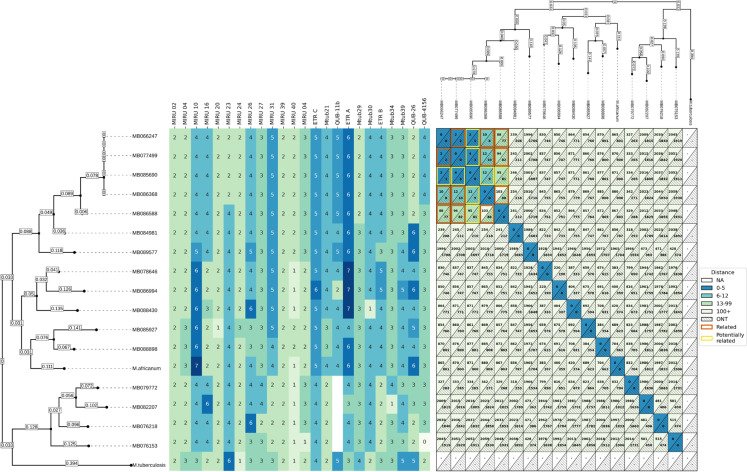
Whole genome sequencing and MIRU-VNTR typing results of *M. africanum* lineage 5 and lineage 6 outbreak. WGS and MIRU-VNTR typing results of *M. africanum* outbreaks, depicting the clustering of lineage 5 (L5) and lineage 6 (L6) isolates. Pairwise SNP distances were calculated using both Illumina (psdm) and Nanopore (TBpore) sequencing methods. Isolates from the same outbreak cluster within the SNP thresholds of ≤5 and ≤12 transmission clusters, aligning with MIRU-VNTR genotyping and epidemiological data. Reference *M. africanum* (ATCC25420) and *M. tuberculosis* (ATCC27294) are included as controls.

**Fig 2:**
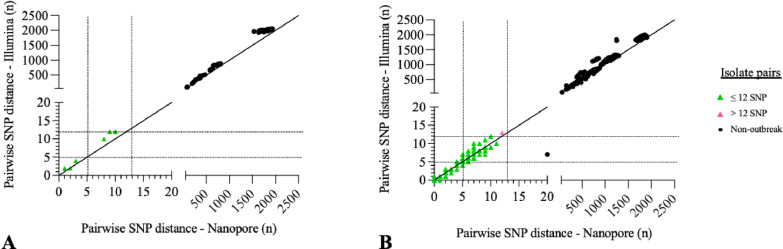
Illumina and Nanopore Whole Genome Sequencing-based clustering comparative analysis. Between isolate pairwise SNP distances using Illumina (COMPASS + psdm; y-axis) and Nanopore (TBpore; x-axis) data for WGS-based typing of *M. africanum* (panel A) and *M. tuberculosis* (panel B) retrospective outbreak isolates. Isolate pairs with identical MIRU-types (triangles) either fall below (green) or above (pink) the 12 SNPs threshold.

The TBpore (Nanopore) and psdm (Illumina) derived SNP distances matrices showed a high level of agreement (Fig 1, S1 in S1 File). For the *M. africanum* outbreak, all 4 isolates previously clustered by MIRU-VNTR and conventional epidemiological investigation remained within the established thresholds when analyzed with both sequencing methods. Although one of those four genotypically clustered isolates (MB085690) was not as strongly related to the others according to public health investigation, all three typing approaches suggested its molecular relatedness. Similarly with the *M. tuberculosis* outbreak and control isolates, all pairwise comparison between the 21 isolates with matching MIRU-types fell within the established SNP threshold when analyzed with both methods, except for one isolate pair (MB086444, L00208036) (Fig 2, S1 in S1 File). This pair exceeded the established threshold by one SNP (13 SNP distance) when sequenced with Illumina and analyzed using COMPASS + psdm, but fell within the 12-SNP threshold when sequenced using the Nanopore platform and TBpore cluster pipeline. Except for one isolate pair (MB090711, MB090710), all *M. tuberculosis* control isolates were shown to be genotypically unrelated to each other as well as with isolates from the known person-to-person transmission event (Figs 1 & 2, S1 in S1 File). Those two isolates had distinct MIRU-VNTR profile and had not been identified as a potential domestic transmission event by public health teams but were respectively 7 and 20 SNP appart when sequenced and analysed with Illumina and ONT platforms and pipelines.

In the *M. africanum* outbreak and control isolates dataset, the Illumina SNP distances ranged from 2 to 2059, while Nanopore’s ranged from 1 to 1931 (Fig 2). The absolute differences in pairwise SNP distances between Illumina and Nanopore ranged from 0 to 432 SNPs, with an average absolute difference of 127 SNPs. Using a paired permutation statistical test and a significance threshold of 0.05, the pairwise SNP distances were found to be significantly higher with Illumina sequencing and the COMPASS analysis pipeline. Similar observations were made with the *M. tuberculosis* data (see S2 in S1 File).

## Discussion

WGS-based molecular typing is increasingly used by public health departments due to the comprehensive insights into TB transmission dynamics it provides as well as its increasing affordability and turnaround time. The diversification and democratization of sequencing technologies and analysis pipelines is ongoing and will help better address the specific needs of different settings. ONT platform acquisition costs are lower than those of Illumina, and ONT protocols and workflows are better suited for smaller sample processing volumes. ONT may hence represent a better strategy for laboratories and public health programs in limited resource settings or those with low TB incidence without other sequencing needs. The impact of those technology-specific relative advantages remains underexplored and merit further evaluation once WGS-based molecular investigations will be performed at scale in reference laboratory and public health workflows including those of resource-limitted settings.

This study confirms the ability of ONT sequencing coupled with the novel open access TBpore pipeline to successfully support molecular epidemiology investigations. This was established using well characterized clinical isolates from two and three distinct outbreaks and sublineages (L4, L5, L6) of the *M. tuberculosis* complex with MIRU and Illumina WGS molecular typing results. Further validation of this novel ONT-based clustering approach on larger datasets with increased geographic catchment, lineage diversity and robust epidemiological metadata should be performed to confirm these results.

Compared to the established standard Illumina NGS platforms, we observed a trend towards lower between isolates SNP distances using ONT and TBpore clusters although, except in one case, this did not affect isolates clustering when using previously accepted transmission thresholds [8,9]. Those between isolates SNP distances differences can be due to the underlying sequencing technology, data pre-processing, processing pipelines, or variant calling parameters [5,10]. Although this finding should also be validated in larger international datasets including samples with more diverse epidemiological and genomic backgrounds, it is consistent with previous work on the validation of Nanopore technology for TB genomic clustering analyses in which this platform was also found to cluster more isolates together [5]. In this previous work, the SNP calling features and clustering pipeline were not the same as those included in TBpore clusters which we evaluated in this study. TBPore uses a more focused decontamination database and optimises variant calling parameters to allow execution of the tool on a standard laptop hence increasing portability of the Nanopore companion analysis pipeline. The previously published analysis used a Nanopore-adapted pipeline which was tailored to a high-performance computing environment where resources were not limiting. Also, clinical epidemiology data from field investigations was not available to contextualize between-sequencing technologies discrepancies.

In our study, the ONT platform and novel pipeline led to the clustering of two isolates which would not have been linked together using standard relatedness thresholds and Illumina technology. Field epidemiology data from public health authorities confirmed that those two isolates were indeed clinically related. Those two isolates also shared the same MIRU-type. Conversely, one pair of isolate was clustered together using Illumina technology but the two isolate did not share an identical MIRU-type and patients had not been identified as being epidemiology related by public health investigations. This potential increased ability of ONT to accurately identify clinically related isolates using previously proposed SNP distance thresholds should be confirmed on larger TB WGS datasets with robust epidemiological metadata.

## Methods

### Sample selection

Clinical isolates included in this study were retrospectively obtained from the Québec Public Health Laboratory (LSPQ). Isolates were purposively included from two distinct domestic TB outbreaks which had previously been characterized by public health investigations and MIRU-VNTR typing [7]. MIRU-VNTR typing was performed at the Canadian National Microbiology Laboratory (LMN). Case isolates (*M. africanum*; n = 4, *M. tuberculosis*; n = 21) were epidemiologically related and control isolates (*M. africanum*; n = 12, *M. tuberculosis*; n = 21) were not. All control isolates were matched with cases based on geographic (administrative region) and temporal (year of sampling) criteria. Reference *M. africanum* (ATCC 25420) and *M.tuberculosis* H37Rv (ATCC 27294) strains were also included as controls for the bioinformatics analyses.

### Culture, extraction, DNA sequencing and quality control

Culture and DNA extraction were performed as per standard protocols on MGIT liquid culture media systems (Becton Dickinson, Franklin Lakes, NJ, USA). All isolates were sequenced with both Illumina and ONT platform from the same DNA extractions. Extracts were sequenced on the Illumina NextSeq500 platform at the Institute for Research in Immunology and Cancer (IRIC). A Paired-End library (Kapa Hyperprep Plus) was prepared on-site following the manufacturer’s instructions and loaded onto a Mid Output flow cell. ONT sequencing libraries were prepared using the ligation sequencing kit 1D (SQK-LSK109) and the Native Barcoding kits 1D (EXP-NBD104 and EXP-NBD114) following the manufacturer’s instructions. The libraries were sequenced on R9.4.1 flow cells on the MinION platform. Detailed sequencing results metrics including genomic coverage are presented as S3 in S1 File.

### Molecular typing and phylogenetic analyses

The Illumina FASTQ data had contaminant (non-*M. tuberculosis*) reads removed using Clockwork [11]. Decontaminated FASTQ files were used to identify genomic single nucleotide polymoprmisms (SNP) and to generate the consensus sequences with CompassCompact (V1.0.2). Briefly, CompassCompact maps reads to the reference with Stampy and calls SNPs with samtools and bcftools. A pairwise distance matrix was created using psdm (V0.1.0) [12]. Nanopore fast5 data were basecalled, filtered, and demultiplexed using Guppy software (V6.1.5) using the super-accuracy model. The resulting FASTQ files were then processed through TBpore (V0.6.0) subcommand *process* in order to characterize the phylogenetic lineage, call variants and generate consensus sequences [5,13]. This removes contamination from the reads using minimap2 against a reduced version of the database used by Clockwork above. It then maps the reads to the reference using minimap2 and calls variants using bcftools. Exact variant filters were previously described in Hall *et al.* [4]. The consensus sequence was obtained by applying all SNPs that passed filtering to the reference genome and masking those that failed filtering (replace with N). The distance between isolates was determined by the consensus sequences using subcommand *cluster*, which uses psdm to generate a SNP distance matrix from the Nanopore consensus sequences. In both ONT and Illumina pipelines, *M. tuberculosis* H37Rv (NCBI NC000962.3) was used as reference for alignment and SNP calling. Lineages were called for both Illumina and Nanopore data using Mykrobe, which uses the lineage-specific SNP markers as previously described [14].

### Clustering comparative analysis

A pairwise distance matrix was used to compare SNP distances between isolates for both NGS technologies. As per previously proposed thresholds, all pairs of isolates having an SNP distance within 5 and 12 were respectively considered as probable or possible transmission clusters [8]. Those clustering results were compared with the MIRU-VNTR genotyping data and contextualized with insights from conventional epidemiological investigation [7]. A paired permutation statistical test was used to assess the statistical significance of the observed differences in SNP distances between technologies. This test was selected as the SNP distance differences between the two technologies represent dependent and paired data points which are not distributed symmetrically around their distribution median.

### Statistical analyses

All statistical analyses were performed using R software V 4.3.1.

### Ethics

This study was approved by the CRCHUM Institutional Review Board (#2020-8441,19.345). This study was performed on retrospective archived samples. Authors could not identify individual participants.

## Supporting information

S1 File Supporting information 1. MIRU-VNTR and WGS typing results of the M. tuberculosis subsp. tuberculosis lineage 4 outbreak. Supporting information 2. Statistical Analysis of Illumina Data Compared to Nanopore Data. Supporting information 3. Illumina and Nanopore Sequencing Performance Metrics.
(DOCX)
