# Supplementary material for: TBpore cluster: A novel phylogenetic pipeline for tuberculosis transmission studies using nanopore next-generation sequencing data
Source: PLoS One. 2025 Jun 16;20(6):e0325914. doi: 10.1371/journal.pone.0325914 (PMC12169557; doi:10.1371/journal.pone.0325914)
Supplement: S1 File — Supporting information 1. MIRU-VNTR and WGS typing results of the M. tuberculosis subsp. tuberculosis lineage 4 outbreak. Supporting information 2. Statistical Analysis of Illumina Data Compared to Nanopore Data. Supporting information 3. Illumina and Nanopore Sequencing Performance Metrics. (DOCX) [file pone.0325914.s001.docx]

# SUPPORTING INFORMATION

##
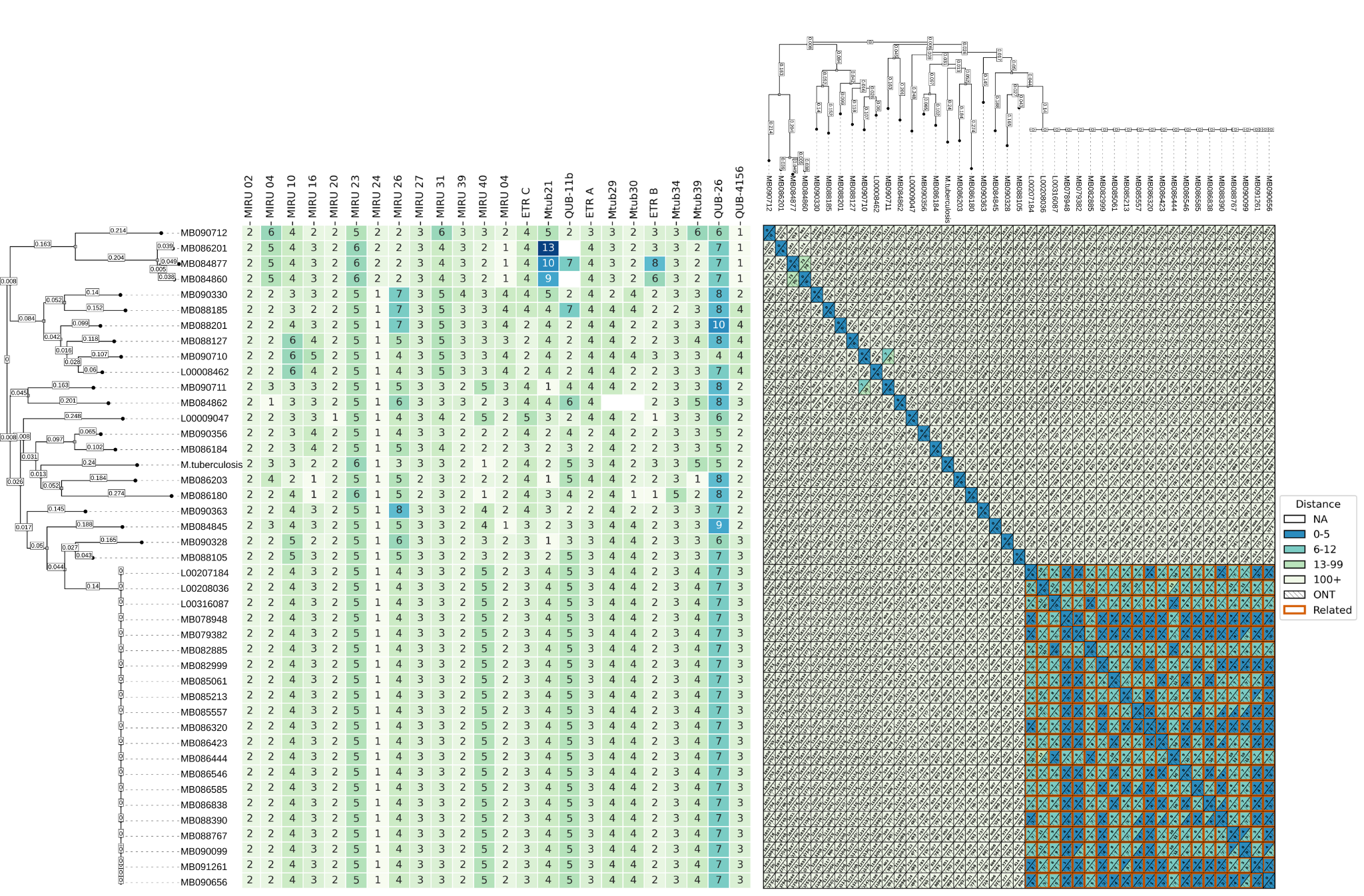
Supporting information 1 - MIRU-VNTR and WGS typing results of the *M. tuberculosis* subsp. *tuberculosis* lineage 4 outbreak

## Supporting information 2 – Statistical Analysis of Illumina Data Compared to Nanopore Data

*M.africanum* statistic summary:

Verification of Variables

The format of the variables was verified using the str(data) command. The results show that all variables are correctly formatted:

## tibble [136 × 3] (S3: tbl_df/tbl/data.frame)

## $ Pair_ID : num [1:136] 1 2 3 4 5 6 7 8 9 10 ...

## $ Illumina_SNP_distances: num [1:136] 704 689 384 420 708 692 383 674 659 387 ...

## $ Nanopore_SNP_distances: num [1:136] 663 623 354 374 631 594 357 615 576 333 ...

Descriptive Analyses

SNP distances obtained using the Illumina technology range from 2 to 2059, while those obtained using the Nanopore technology range from 1 to 1931. The absolute differences between SNP distances from Illumina and Nanopore range from 0 to 432 SNPs, with a mean absolute difference of 127 SNPs.

Correlation Analysis (Scatter Plot)

ggplot(data, aes(x = Illumina_SNP_distances, y = Nanopore_SNP_distances)) +

geom_point() +

geom_abline(slope = 1, intercept = 0, linetype = "dashed", color = "red")


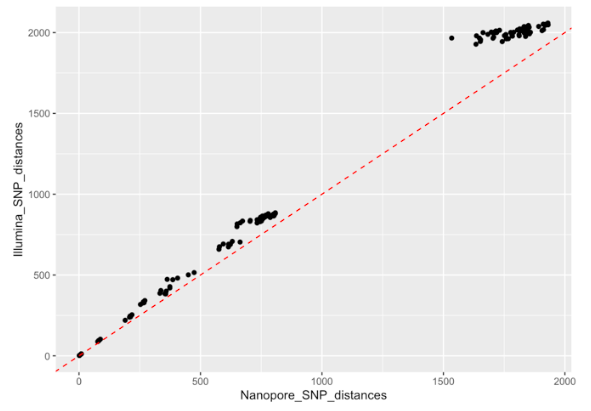


The red line represents the line of equality. The data show a generally linear correlation but are divided into two groups. The correlation coefficient is 0.9972624.

Comparative Analysis (Boxplots)

A boxplot was created to compare SNP distances by sequencing tool:

ggplot(long_data, aes(x = Sequencing_tool, y = SNP_distance)) +

geom_boxplot() +

geom_jitter(width = 0.1) +

labs(title = "SNP Distances by sequencing tool", x = "Sequencing tool", y = "SNP Distance") +

theme_minimal()


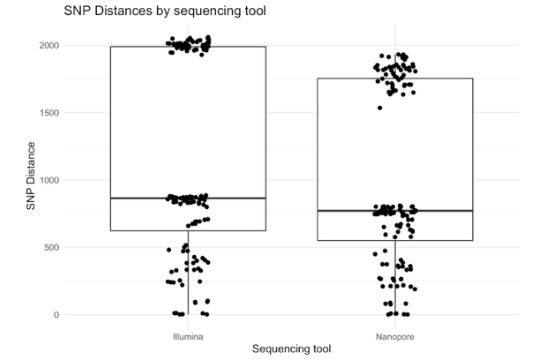


Another boxplot was generated to visualize the distribution of differences between distances obtained with Illumina and Nanopore:

data$differences <- data$Illumina_SNP_distances-data$Nanopore_SNP_distances

ggplot(data, aes(x = "", y = differences)) +

geom_boxplot() +

geom_jitter(width = 0.1) +

labs(title = "Distribution of differences (Illumina - Nanopore)", y = "Differences", x = "") +

theme_minimal()


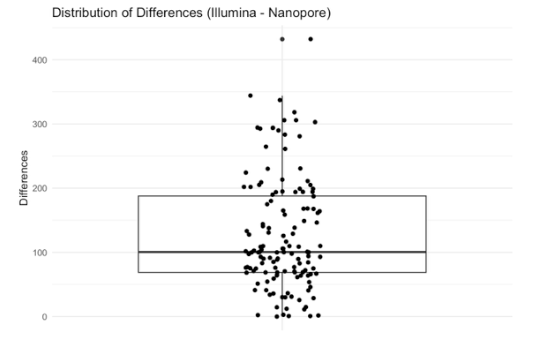


The analysis shows that the distribution of differences is not symmetrical around the median, with a skew toward positive values. Due to this asymmetry, a permutation test was used instead of a Wilcoxon signed-rank test.

Statistical Analyses

A permutation test was conducted due to the non-independence of the paired data (each patient appearing in multiple pairs) and the asymmetry of the differences around the median.

The test was conducted with 10,000 permutations:

set.seed(123)

n_permutations <- 10000

perm_stats <- replicate(n_permutations, { sign_flips <- sample(c(1, 1),length(data$Illumina_SNP_distances), replace = TRUE)

permuted_differences <- sign_flips *(data$Illumina_SNP_distances - data$Nanopore_SNP_distances) mean(permuted_differences)

 })

hist(perm_stats)

abline(v = observed_statistic, col = "red", lwd = 2)


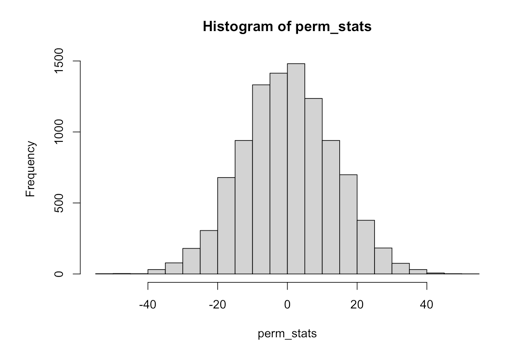


The test produced an observed statistic of 126.8235, with a p-value of 0, indicating that the difference in SNP distances between the two methods is statistically significant.

Alternative Permutation Method

An alternative method using the Broman package was employed to confirm the results.

library(broman) paired. perm.test(data$differences, n.perm = 10000, pval = TRUE)

The p-value from this method was also 0.

Conclusion

The permutation test revealed a statistically significant difference in SNP distances between the Illumina and Nanopore sequencing methods (p < 0.05). Due to the paired structure of the data and the asymmetry in the distribution of differences, the permutation test was deemed the most appropriate analytical approach.

Assumption Verification

The assumptions necessary for the validity of the permutation test were verified:

- Exchangeability: Satisfied, as samples assigned to one sequencing technology could have been used for the other.

*M. tuberculosis* statistic summary:

Verification of Variables

The format of the variables was verified using the str(data) command. The results show that all variables are correctly formatted:

## tibble [903 × 3] (S3: tbl_df/tbl/data.frame)

## $ Pair_ID : num [1:903] 1 2 3 4 5 6 7 8 9 10 ...

## $ Illumina_SNP_distances: num [1:903] 1273 637 845 847 853 ...

## $ Nanopore_SNP_distances: num [1:903] 1181 418 778 785 813 ...

Descriptive Analyses

SNP distances obtained using the Illumina technology range from 0 to 2004, while those obtained using the Nanopore technology range from 0 to 1905. The absolute differences between SNP distances from Illumina and Nanopore range from 0 to 595 SNPs, with a mean absolute difference of 59 SNPs.

Correlation Analysis (Scatter Plot)

ggplot(data, aes(x = Illumina_SNP_distances, y = Nanopore_SNP_distances)) +

geom_point() +

geom_abline(slope = 1, intercept = 0, linetype = "dashed", color = "red")


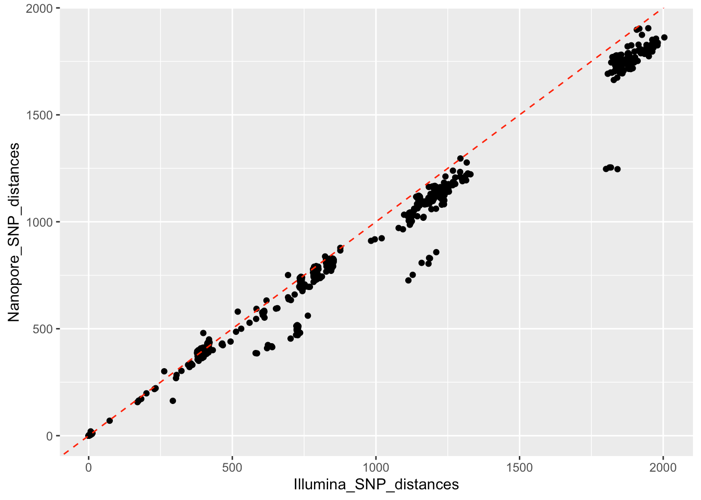


The red line represents the line of equality. The data show a generally linear correlation with a correlation coefficient of 0.9952693.

Comparative Analysis (Boxplots)

A boxplot was created to compare SNP distances by sequencing tool:

ggplot(long_data, aes(x = Sequencing_tool, y = SNP_distance)) +

geom_boxplot() +

geom_jitter(width = 0.1) +

labs(title = "SNP Distances by sequencing tool", x = "Sequencing tool", y = "SNP Distance") +

theme_minimal()


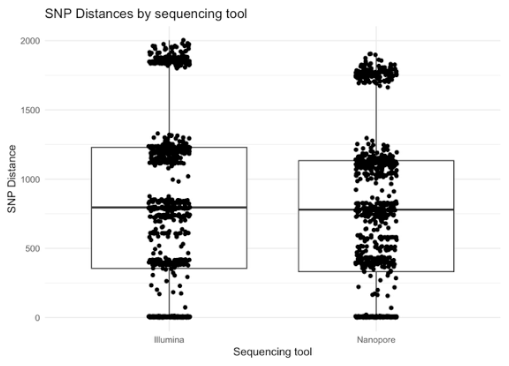


Another boxplot was generated to visualize the distribution of differences between distances obtained with Illumina and Nanopore:

data$differences <- data$Illumina_SNP_distances-data$Nanopore_SNP_distances

ggplot(data, aes(x = "", y = differences)) +

geom_boxplot() +

geom_jitter(width = 0.1) +

labs(title = "Distribution of differences (Illumina - Nanopore)", y = "Differences", x = "") +

theme_minimal()


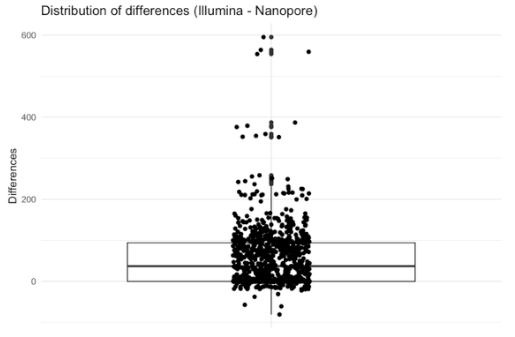


The analysis shows that the distribution of differences is not symmetrical around the median, with a skew toward positive values. Due to this asymmetry, a permutation test was used instead of a Wilcoxon signed-rank test.

Statistical Analyses

A permutation test was conducted due to the non-independence of the paired data (each patient appearing in multiple pairs) and the asymmetry of the differences around the median.

The test was conducted with 10,000 permutations:

set.seed(123)

n_permutations <- 10000

perm_stats <- replicate(n_permutations, {

 sign_flips <- sample(c(1, 1), length(data$Illumina_SNP_distances), replace =TRUE)

permuted_differences <- sign_flips * (data$Illumina_SNP_distances - data$Nanopore_SNP_distances)mean(permuted_differences)

})

hist(perm_stats)

abline(v = observed_statistic, col = "red", lwd = 2)


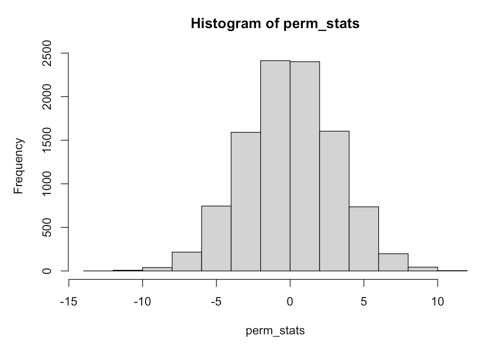


The test produced an observed statistic of 57.2392, with a p-value of 0, indicating that the difference in SNP distances between the two methods is statistically significant.

Alternative Permutation Method

An alternative method using the Broman package was employed to confirm the results.

library(broman) paired. perm.test(data$differences, n.perm = 10000, pval = TRUE)

The p-value from this method was also 0.

Conclusion

Given the paired nature of the data (Illumina-Nanopore) and the asymmetry in the distribution of differences, a permutation test was selected as the most appropriate method. The null hypothesis, which posited that the observed differences between the two sequencing methods were due to random variation, was rejected. The findings demonstrate a statistically significant difference in SNP distances between the methods (p < 0.05).

Assumption Verification

The assumptions necessary for the validity of the permutation test were verified:

- Exchangeability: Satisfied, as samples assigned to one sequencing technology could have been used for the other.

## Supporting information 3 –Illumina and Nanopore Sequencing Performance Metrics

|  | Nanopore | | | Illumina | | |
| --- | --- | --- | --- | --- | --- | --- |
| *M.africanum* Samples | Numbers of reads | Median depth | % Genome coverage | Numbers of reads | Median depth | % Genome coverage |
| ATCC_25420 | 3094,28 | 37 | 99.571 | 22,600,608 | 281 | 99.656 |
| MB076153 | 2040,85 | 83 | 99.649 | 26,063,530 | 307 | 99.645 |
| MB076218 | 7226,2 | 41 | 99.581 | 23,475,134 | 282 | 99.598 |
| MB078646 | 1159,54 | 85 | 99.418 | 20,408,904 | 253 | 99.402 |
| MB079772 | 3384,1 | 11 | 99.528 | 23,714,368 | 291.0 | 99.761 |
| MB082207 | 4862,7 | 16 | 99.302 | 23,055,524 | 270 | 99.629 |
| MB084981 | 5171 | 19 | 99.288 | 21,735,036 | 268.0 | 99.429 |
| MB066247 (case) | 1055,27 | 38 | 99.201 | 24,368,944 | 304 | 99.287 |
| MB077499 (case) | 1416,75 | 84 | 99.197 | 27,619,616 | 346.0 | 99.265 |
| MB086368 (case) | 2489,61 | 81 | 99.54 | 21,384,734 | 266 | 99.364 |
| MB085690 (case) | 1619,13 | 70 | 99.198 | 20,003,186 | 251 | 99.297 |
| MB085927 | 1419,66 | 85 | 99.586 | 21,561,060 | 271 | 99.734 |
| MB086588 | 6302,1 | 26 | 99.425 | 22,455,426 | 274.0 | 99.544 |
| MB086994 | 5652,1 | 14 | 99.392 | 18,808,420 | 236.0 | 99.553 |
| MB088430 | 6151,8 | 25 | 99.365 | 20,739,628 | 259.0 | 99.462 |
| MB088898 | 2047,64 | 83 | 99.684 | 21,353,858 | 262 | 99.619 |
| MB089577 | 1903,59 | 83 | 99.71 | 21,230,046 | 266 | 99.737 |

|  | Nanopore | | | Illumina | | |
| --- | --- | --- | --- | --- | --- | --- |
| *M.tuberculosis* Samples | Numbers of reads | Mean coverage | % Genome coverage | Numbers of reads | Mean coverage | % Genome coverage |
| ATCC27294 | 462024 | 45 | 99.627 | 7,438,058 | 72 | 99.7 |
| L00008462 | 254580 | 31 | 99.584 | 7,398,750 | 69 | 99.698 |
| L00009047 | 53260 | 6 | 98.937 | 8,452,440 | 86.0 | 99.635 |
| MB084845 | 137287 | 54 | 99.519 | 6,905,242 | 74.0 | 99.598 |
| MB084860 | 361121 | 114 | 99.681 | 8,149,946 | 87 | 99.676 |
| MB084862 | 217769 | 100 | 99.693 | 7,821,996 | 85 | 99.68 |
| MB084877 | 324840 | 100 | 99.657 | 7,169,194 | 75 | 99.658 |
| MB086180 | 246646 | 119 | 99.711 | 7,712,906 | 84.0 | 99.704 |
| MB086184 | 333009 | 116 | 99.543 | 6,772,444 | 73 | 99.532 |
| MB086201 | 371304 | 115 | 99.67 | 6,822,990 | 75 | 99.57 |
| MB086203 | 323060 | 117 | 99.537 | 6,568,532 | 71 | 99.658 |
| L00207184 (case) | 266608 | 27 | 99.587 | 9,581,866 | 91.0 | 99.764 |
| L00208036 (case) | 114339 | 12 | 99.555 | 6,661,664 | 66.0 | 99.718 |
| L00316087 (case) | 567598 | 52 | 99.637 | 7,710,502 | 75.0 | 99.706 |
| MB078948 (case) | 178426 | 68 | 99.636 | 7,910,792 | 85 | 99.677 |
| MB079382 (case) | 261046 | 96 | 99.68 | 7,863,140 | 86.0 | 99.699 |
| MB082885 (case) | 144441 | 55 | 99.599 | 6,931,076 | 75.0 | 99.703 |
| MB082999 (case) | 259299 | 99 | 99.638 | 8,314,920 | 90.0 | 99.725 |
| MB085061 (case) | 271718 | 116 | 99.7 | 7,051,130 | 76.0 | 99.718 |
| MB085213 (case) | 245651 | 105 | 99.711 | 7,059,672 | 77.0 | 99.714 |
| MB085557 (case) | 170060 | 77 | 99.668 | 7,739,132 | 85.0 | 99.696 |
| MB086320 (case) | 356558 | 114 | 99.714 | 8,084,960 | 87.0 | 99.721 |
| MB086423 (case) | 309284 | 116 | 99.697 | 6,555,270 | 71.0 | 99.693 |
| MB086444 (case) | 123738 | 43 | 99.605 | 5,705,860 | 62 | 99.645 |
| MB086546 (case) | 214698 | 80 | 99.647 | 6,597,240 | 70.0 | 99.704 |
| MB086585 (case) | 239360 | 88 | 99.679 | 5,729,070 | 62.0 | 99.675 |
| MB086838 (case) | 151307 | 57 | 99.658 | 6,018,512 | 65.0 | 99.685 |
| MB088390 (case) | 257094 | 24 | 99.632 | 7,517,586 | 76.0 | 99.719 |
| MB088767 (case) | 200730 | 15 | 99.542 | 7,406,474 | 77.0 | 99.718 |
| MB090099 (case) | 165429 | 13 | 99.555 | 6,702,834 | 73.0 | 99.7 |
| MB090656 (case) | 236162 | 26 | 99.601 | 8,212,146 | 82 | 99.68 |
| MB091261 (case) | 136628 | 15 | 99.565 | 7,788,358 | 74.0 | 99.732 |
| MB088105 | 295632 | 117 | 99.65 | 6,320,150 | 69.0 | 99.63 |
| MB088127 | 592910 | 56 | 99.627 | 8,187,476 | 82.0 | 99.523 |
| MB088185 | 236781 | 20 | 99.443 | 7,459,498 | 78 | 99.555 |
| MB088201 | 943811 | 71 | 99.697 | 7,900,704 | 82.0 | 99.707 |
| MB090328 | 434938 | 39 | 99.605 | 6,831,504 | 69.0 | 99.717 |
| MB090330 | 243215 | 30 | 99.588 | 7,084,536 | 70.0 | 99.715 |
| MB090356 | 355423 | 40 | 99.509 | 7,972,740 | 77.0 | 99.633 |
| MB090363 | 238354 | 25 | 99.297 | 8,349,116 | 83 | 99.685 |
| MB090710 | 361334 | 38 | 99.55 | 7,658,918 | 70 | 99.43 |
| MB090711 | 866249 | 63 | 99.707 | 7,318,292 | 70.0 | 99.696 |
| MB090712 | 452752 | 40 | 99.397 | 8,164,074 | 79 | 99.572 |
